# Amphibian-Derived Peptide Analog TB_KKG6K: A Powerful Drug Candidate Against *Candida albicans* with Anti-Biofilm Efficacy

**DOI:** 10.3390/jof12010011

**Published:** 2025-12-23

**Authors:** Cristina Schöpf, Anik Geschwindt, Magdalena Knapp, Anna C. Seybold, Débora C. Coraça-Huber, Michael J. Ausserlechner, Alessandra Romanelli, Florentine Marx

**Affiliations:** 1 Institute of Molecular Biology, Biocenter, Medical University of Innsbruck, 6020 Innsbruck, Austria; cristina.schoepf@i-med.ac.at (C.S.); anik.geschwindt@gmail.com (A.G.); 2 Department of Zoology, University of Innsbruck, 6020 Innsbruck, Austria; magdalena.knapp@uibk.ac.at (M.K.); anna.seybold@uibk.ac.at (A.C.S.); 3 Research Laboratory for Implant Associated Infections (BIOFILM LAB), Experimental Orthopaedics, University Hospital for Orthopaedics and Traumatology, Medical University of Innsbruck, 6020 Innsbruck, Austria; debora.coraca-huber@i-med.ac.at; 4 3D Bioprinting Core Facility, Department of Pediatrics I, Medical University of Innsbruck, 6020 Innsbruck, Austria; michael.j.ausserlechner@i-med.ac.at; 5 Department of Pharmaceutical Sciences, University of Milan, 20133 Milan, Italy

**Keywords:** antifungal, biofilm, *Candida albicans*, in vitro, fungicidal, resistance, silicone, temporin B analog

## Abstract

*Candida albicans*, a commensal and opportunistic fungal pathogen, is a major clinical concern due to its ability to cause infections ranging from mild mucosal conditions to life-threatening systemic diseases, particularly in immunocompromised patients. Its capacity to form biofilms on medical devices further complicates treatment by enhancing antifungal resistance and immune evasion. In the search for novel therapeutic strategies, the lysine-enriched amphibian-derived temporin B analog, TB_KKG6K, has emerged as a promising antifungal agent. This study demonstrates that TB_KKG6K exhibits potent fungicidal activity against planktonic *C. albicans* cells, with a low potential to induce adaptation or resistance. TB_KKG6K has no adverse impact on the anti-*Candida* efficacy of standard antifungal drugs when applied in combination, interacting additively with amphotericin B and caspofungin in a fungicidal mode of action. Additionally, TB_KKG6K effectively reduces biofilm maturation on silicone elastomers, a material commonly used in medical devices, further highlighting its therapeutic potential. These data together with our previous documentation of minimal cytotoxicity and irritation potential in human cells makes TB_KKG6K a strong candidate for combating both planktonic and biofilm-associated *C. albicans* infections. These findings underscore the dual efficacy of TB_KKG6K and its potential to address the challenges posed by *C. albicans* in clinical settings.

## 1. Introduction

*Candida albicans* is a commensal and opportunistic pathogen commonly found within the human microbiota. It is capable of forming complex biofilms, in which different cell types (yeast-like cells, germinating cells, pseudohyphae and hyphae) are embedded in a protective extracellular matrix (ECM), composed of glycoproteins, polysaccharides, lipids and nucleic acids [1,2,3]. *C. albicans* biofilms are associated with a range of clinical manifestations, from mild local infections to life-threatening conditions such as candidemia, which may result in high mortality rates in patients with comorbidities [4,5,6]. The protective biofilm environment substantially enhances the pathogenic potential of *C. albicans* by promoting resistance to antifungal drugs and evasion of host immune defenses, a matter of significant concern within the clinical context [1,7,8,9].

Biofilm formation on abiotic surfaces is a major factor in device-associated complication in the clinics where *C. albicans* causes persistent hospital-acquired infections resistant to antifungal treatment, and compromises the functionality of implanted medical devices, necessitating premature replacement and contributing to escalating healthcare costs [10,11,12,13]. *C. albicans* is able to form biofilms on almost any type of medical equipment, including systemic and topical devices, such as contact lenses and dentures, and those that come into contact with or traverse the skin, particularly synthetic polymers such as silicone found in catheters or prostheses [1,8,14]. Adherence of *C. albicans* to silicone and subsequent biofilm formation has been shown to reduce the efficacy of standard antifungal drugs like azoles, polyenes or echinocandins, due to the development of resistance mechanisms [15,16,17]. This situation highlights the necessity to search for new antifungal molecules that are effective against yeast biofilms.

Antimicrobial peptides (AMPs) have been identified as promising candidates for the development of novel antimicrobial therapeutic agents. The European red frog *Rana temporaria* secretes the AMPs temporins by granular glands to protect their skin from infection with microbial pathogens. These short (8–14 amino acid long), mildly cationic (0 to +3 at pH 7) peptides belong to one of the biggest AMP families in nature [18]. Rational design and chemical modifications enabled the creation of peptide analogs with different primary structures and improved efficacy in comparison to their parent peptides. One promising example is represented by the analog of temporin B (TB), named TB_KKG6K (KKLLPIVKNLLKSLL; molecular weight [MW]; 1718.2 Da). This lysine-enriched TB analog exhibits an increased antimicrobial spectrum and enhanced tolerability in the host compared to TB [19]. We have recently been able to show that this peptide analog acts in a fungicidal way against planktonic and sessile *C. albicans* cells in vitro. Its mode of action affects the cell membrane function, induces the production of intracellular reactive oxygen species (iROS) and results in the disintegration of the subcellular structures in the yeast cells, while showing low cytotoxicity in human primary cells and low irritation potential in three-dimensional (3D) reconstructed human skin [20,21,22].

In the present study we wanted to investigate in more detail the anti-*Candida* efficacy of TB_KKG6K. By combining microbiological and molecular biology analyses, and high-end microscopy, we collected data which for the first time provide evidence for the low potential of TB_KKG6K to induce adaptation or resistance in planktonic *C. albicans* cells, its additive interaction with the standard drugs amphotericin B and caspofungin, respectively, and a comprehensive insight into its efficacy to reduce biofilm maturation on the surface of a medically relevant silicone elastomer.

## 2. Material and Methods

If not otherwise stated, the chemicals and compounds used in this study were purchased from Sigma-Aldrich, St. Louis, MO, USA. All media and solutions used are summarized in Table 1.

### 2.1. Peptide Synthesis

TB_KKG6K was synthesized and purified by reversed-phase high performance liquid chromatography (RP-HPLC) as described previously [21].

### 2.2. Drug Susceptibility Testing

The susceptibility of *C. albicans* CBS 5982 for antifungals was determined by broth microdilution assays, which were conducted in flat bottom 96-well microtiter plates (Nunclon Delta, Thermo Fisher Scientific, Waltham, MA, USA) as described earlier [20,23]. An overnight culture of *C. albicans* was washed and adjusted to 1 × 10^4^ cells mL^−1^ in 0.05× PDB (Carl Roth, Karlsruhe, Germany). One-hundred µL of this cell suspension was combined with 100 µL of two-fold serial dilutions of the test compounds prepared in the same medium. Antifungals were tested against *C. albicans* in the following concentration ranges: TB_KKG6K (0.25–64.0 µM), amphotericin B (0.002–4.33 µM), caspofungin (Santa Cruz Biotechnology, Dallas, TX, USA; 0.001–0.41 µM), fluconazole (0.05–26.1 µM), and 5-flucytosine (5-FC; TCI Deutschland GmbH, Eschborn, Germany; 0.06–31.0 µM). The plates were incubated at 30 °C for 24–48 h under static condition and growth was assessed spectrophotometrically at wavelength (λ) 620 nm in a multimode plate reader (FLUOstar Omega, BMG Labtech, Ortenberg, Germany). The OD_620_ value of the untreated control was set to represent 100% growth. The results were expressed as the percentage of growth relative to the untreated control. The minimal inhibitory concentration 90 (MIC_90_) of the antifungal compounds was defined as the lowest concentration that showed ≥90% growth reduction compared to the untreated control. For the determination of the minimal fungicidal concentration (MFC) of TB_KKG6K and amphotericin B, the same experimental conditions as described above were used. After 24 h and 48 h of incubation, respectively, the content of the wells at or above the MIC_90_ were plated on PDA and incubated at 30 °C for another 24 h to count the colony forming units (CFU). The MFC was defined as the lowest concentration of a compound at which no fungal growth could be detected. Experiments were repeated at least twice.

### 2.3. Checkerboard Assay

The potential of TB_KKG6K to inhibit *C. albicans* growth in combination with amphotericin B, fluconazole, caspofungin, and 5-FC, was evaluated using the checkerboard method based on the broth microdilution technique as previously described [20,24]. The assays were performed in 0.05× PDB. TB_KKG6K was tested starting at twice its respective MIC_90_ value, and then serially diluted to lower concentrations (concentration range tested 0.06–4 µM). The licensed antifungals were prepared as described in Section 2.2. One antifungal was diluted along the X-axis (columns) and the second along the Y-axis (rows), creating a matrix of unique combinations of drug concentrations. A 50 μL aliquot of each four-fold concentrated antifungal compound, combined with 50 μL of the second four-fold concentrated compound or 50 μL of medium (control for single compound testing), was dispensed into a 96-well microplate and mixed with 100 μL of a 10^4^ cells mL^−1^ inoculum, resulting in a final volume of 200 μL per well. The microplates were incubated statically at 30 °C for 24–48 h, and growth was assessed visually and spectrophotometrically at 620 nm. The OD_620_ value of the untreated control was set to represent 100% growth. The results were expressed as the percentage of growth relative to the untreated control.

Antifungal interactions of the individual drug combinations were analyzed using the fractional inhibitory concentration index (FICI) as described [24,25]. The FICI was calculated by summing the fractional inhibitory concentrations (FICs) for each compound, based on the MIC_90_ values of the individual antifungals (A and B) both in combination and individually: ΣFIC = FIC_A_ + FIC_B_. FIC_A_ refers to the MIC_90_ of drug A in combination/MIC_90_ of drug A alone and FIC_B_ to the MIC_90_ of drug B in combination/MIC_90_ of drug B alone. FICI was defined as the lowest ΣFIC determined in three independent experiments. Interactions were classified synergistic (FICI ≤ 0.5), additive (0.5 < FICI ≤ 1), indifferent (1 < FICI < 4), and antagonistic (FICI > 4) [24]. Experiments were repeated three times (n = 3). The MFC of antifungal combinations showing an additive interaction was assessed as described above.

### 2.4. Drug Adaptation Experiment

A drug adaptation experiment was performed in vitro according to [26] with slight modifications [27]. In brief, three individual colonies of *C. albicans* per condition (treatment with TB_KKG6K and fluconazole, respectively, and untreated growth control) were picked from a PDA plate and grown in 0.05× PDB, resulting in three lineages per condition. Then, a suspension of 3 × 10^4^ cells mL^−1^ of *C. albicans* was prepared in 0.05× PDB in triplicate for each lineage. For the growth control, 1 mL of the cell suspension was transferred to 2 mL of drug-free 0.05× PDB medium. For the antifungal treatment, 1 mL of the cell suspension was mixed with 2 mL of 0.05× PDB medium containing half the MIC_90_ (0.5× MIC_90_) of TB_KKG6K (1 µM) or fluconazole (3.2 µM), which served as the positive control for adaptation. The growth control was not exposed to antifungal treatment. All samples were incubated at 30 °C for 24 h and with shaking at 200 rpm in 10 mL test tubes. Then, 100 µL of each sample was transferred to 3 mL of fresh medium, maintaining the 0.5× MIC_90_. This subculturing process was repeated at 24-h intervals for a total of five days. Subsequently, the cells were diluted 1:30 into medium supplemented with the doubled concentration of antifungal compound. Subculturing was continued in 24-h intervals at this antifungal concentration for three days. Then the concentration of the antifungal compound was doubled again. These cultivation cycles were repeated until no cells proliferated any more or an antifungal compound concentration of 32× MIC_90_ was reached. The untreated cells of the growth control were transferred in a 1:30 dilution daily to fresh, but drug-free, 0.05× PDB medium. Throughout the experiment, the growth was monitored spectrophotometrically using a multimode microplate reader by determining the OD_620_ of a 200 µL sample at the end of the third subculturing step of each concentration cycle. When the OD_620_ value dropped to background level (medium without cells), the culture was streaked out onto YPD agar and incubated at 30 °C for 24 h to count the surviving cells (CFU).

### 2.5. C. albicans Biofilm Cultivation on Silicone Elastomer Discs

Discs were laser-cut from silicone sheets (0.25 mm, MVQ Silicones GmbH, Weinheim, Germany) with diameters of 9 mm for 48-well plates (VWR, Randnor, PA, USA) and 14 mm for 24-well plates (CytoOne, Starlab, Hamburg, Germany). They were washed by vortexing in 15 mL of double distilled H_2_O, followed by sonication (35 kHz; Bandelin Sonorex, BANDELIN electronic GmbH & Co. KG, Berlin, Germany) for 10 min. The water was replaced, and this process was repeated twice. Subsequently, the discs were immersed in 15 mL of 70% ethanol for 24 h. Discs were dried under sterile conditions in a safety cabinet and exposed to UV light (λ = 254 nm) on both sides for 30 min each. The discs were stored under sterile conditions at room temperature until usage.

Before seeding *C. albicans* cells, the discs were placed in a 48-well or 24-well plate and covered with heat-inactivated, sterile fetal bovine serum (PAN-Biotech GmbH, Aidenbach, Germany). They were gently shaken at 100 rpm for 10 min and then incubated statically at 37 °C overnight. The next day, the discs were washed with 0.5–1 mL of sterile PBS for 10 min under gentle shaking (100 rpm). The PBS was removed, and the discs were air-dried under sterile conditions before being transferred to a new plate.

Then, 500 µL from a freshly diluted overnight culture of *C. albicans* containing 10^6^ cells mL^−1^ in 0.05× PDB were seeded into each well of a 48-well plate equipped with 9 mm diameter silicone discs, resulting in a cell density of 5 × 10^5^ cells per well. In 24-well plates equipped with 14 mm diameter silicone discs, the cell density and volume were adjusted to the larger surface area. Accordingly, 1.2 mL of a *C. albicans* suspension containing 5 × 10^6^ cells mL^−1^ were seeded into each well, resulting in a cell density of 6 × 10^6^ cells per well. Cells were distributed by moving the suspension in the culture plates in circular motions and then incubated statically at 30 °C for up to 72 h. Biofilm formation was checked microscopically and the medium was exchanged every 24 h to remove non-adherent cells.

### 2.6. Antifungal Therapy of Sessile C. albicans Cells

The anti-*Candida* efficacy of 2–50 µM TB_KKG6K (corresponding to 1×–25× MIC_90_) was tested on a 48-h matured biofilm and compared to untreated growth controls after incubation for 4 h and 24 h at 30 °C. Samples exposed to amphotericin B (1.4 µM; 10× MIC_90_) under the same incubation conditions was included as positive drug controls.

Then the medium was aspirated off, and the discs were placed in a 2 mL reaction tube with 1 mL of sterile PBS. They were vortexed at the highest setting for 30 s, followed by three cycles of sonication (35 kHz) in a water bath (Bandelin Sonorex) for 1 min each as previously described [28]. Vortexing was then repeated. Serial dilutions were prepared, and 100 µL of each dilution was plated on PDA. Plates were incubated for 24 h at 30 °C for the quantification of CFU. Absolute CFU values from individual experiments were log_10_-transformed. The log_10_ difference was calculated by comparing treated samples to the untreated biofilm control within each biological replicate (n = 3) [log_10_(CFU_Control_) − log_10_(CFU_Treatment_)]. Relative survival (%) was determined from the absolute CFU data within each biological replicate (n = 3) as [(CFU_Treatment_/CFU_Control_) × 100].

### 2.7. Scanning Electron Microscopy (SEM)

Discs with *C. albicans* biofilm were harvested by removing the culture medium and transferring them into a new 48-well plate. They were fixed in 2.5% glutaraldehyde (vol/vol in PBS) at 4 °C for 24 h. Dehydration was performed using an ascending ethanol (50-70-80-90%) or acetone series (50-70-80-90-100%). The discs were then mounted on aluminum pins and sputter-coated with gold. Microscopy was performed using JSM-6010LV (JEOL GmbH, Freising, Germany) or TESCAN CLARA SEM (TESCAN GROUP, Brno, Czech Republic). Experiments were repeated three times (n = 3).

### 2.8. Confocal Microscopy

Biofilm samples were fixed on the silicone discs in 500 µL of 4% (wt/vol) paraformaldehyde in PBS for 30 min at room temperature. The fixative was then removed, and the discs were washed with 500 µL of PBS for 10 min. Staining was started with Concanavalin A conjugate (100 µg mL^−1^, Alexa Fluor 633 [AF633], Thermo Fisher Scientific) for 30 min. Then, the biofilm was thoroughly washed with D-PBS and subsequently stained with Calcofluor White (1 mg mL^−1^) for 2 min, before washing the samples again with D-PBS to ensure the removal of any residual stain. Subsequently, the biofilm on the silicone discs was mounted in 7 µL of Fluoroshield™ and covered with a 12 mm high precision coverslip (Thorlabs Inc., Newton, NJ, USA). Biofilm was analyzed using a SP8 gSTED microscope (Leica Microsystems GmbH, Wetzlar, Germany). All recordings were processed using the same parameters in Huygens Professional 25.04 (Scientific Volume Imaging B.V., Hilversum, The Netherlands). Experiments were repeated three times (n = 3).

### 2.9. RNA Extraction and Quantitative PCR

Total RNA was isolated from sessile cells grown on 14 mm silicone discs in 24-well plates. Biofilm that had matured for 48 h was subjected to treatment with 5 µM TB_KKG6K for a period of 4 h. Then, eight discs per condition were pooled for RNA extraction and cells were detached by rigorous pipetting. The disruption of cells was achieved mechanically by employing glass beads (0.50–0.75 mm, RETSCH GmbH, Haan, Germany) in 1 mL of TRI reagent (TRI Reagent^®^). This process was conducted three times in a Mixer Mill (MM400, RETSCH GmbH) for 2 min at a frequency of 30 Hz. The tubes were cooled for 1 min on ice between each repetition. Subsequently, 200 μL of chloroform was added to each tube, and the tubes were vortexed for 15 s and left to incubate at room temperature for 5 min. Then the tubes were centrifuged at 4 °C and 12,000× *g* for 10 min. The upper phase containing the RNA was transferred to a 1.5 mL microcentrifuge tube, and an equal volume of 70% (vol/vol) isopropanol was added. A quantity of 20 µg of glycogen was added to the solution, after which the RNA was precipitated at −20 °C overnight. After centrifugation of the samples at 4 °C and 12,000× *g* for 10 min the isopropanol was removed, and the RNA pellet was washed twice in 500 µL of 75% (vol/vol) ethanol. For elution, 20 μL of RNase-free water was used. Subsequently, the RNA was treated with 2 units of DNAse I (RNase free, New England Biolabs, Inc., Ipswich, MA, USA) per 10 µg of RNA at 37 °C for 30 min. The RNA was purified by phenol-chloroform-isoamyl alcohol (Carl Roth) extraction, and the concentration and quality of RNA were analyzed using a NanoPhotometer™ (NP80, Implen GmbH, Munich, Germany).

One μg of RNA was used for cDNA synthesis (iScriptTM RT Supermix, Bio-Rad Laboratories, Hercules, CA, USA). The reaction was performed using the PikoReal 96 System (PikoReal 96 Realtime PCR System, Thermo Fisher Scientific). The iQTM SYBR Green Supermix kit (Bio-Rad Laboratories) was applied for qPCR, with a total volume of 20 μL per reaction. Experiments were repeated three times (n = 3). The primer pairs listed in Table 2 were selected according to their high primer efficiencies (*ACT1*, 98%; *EFB1*, 99%; *BRG1*, 104%; *FKS1*, 106%), and they produced single, sharp melt peaks within a narrow temperature range, indicating specific and reproducible amplification. For each reaction, 50 ng of cDNA were used. The gene expression fold change was calculated using the ΔΔ Ct values [29].

### 2.10. Statistics

Statistical analysis was conducted using Prism 9.1.0 (GraphPad Software, San Diego, CA, USA). Values are given as mean ± standard deviation (SD) per experimental setting (n = 3) and statistical significance (* *p* ≤ 0.05; ** *p* ≤ 0.005) was determined by one-way ANOVA, followed by Dunnett’s test, if not stated otherwise.

## 3. Results

### 3.1. TB_KKG6K Exhibits Strong Sustained Candidacidal Efficacy

We could previously show that TB_KKG6K acts in a fungicial mode of action on planktonic and sessile growing *C. albicans* cells within 24 h of incubation [20]. In this study, we investigated the effectiveness of the peptide’s antifungal activity in completely eradicating *C. albicans* cells, thereby preventing the possibility of surviving cells resuming growth and forming biofilms during prolonged incubation. To address this, we determined the peptide’s MIC_90_ and MFC after 24 h and 48 h of incubation with *C. albicans*. The results, summarized in Table 3, show that the MFC of TB_KKG6K matched its MIC_90_ (2 μM) at both tested time points, with no *C. albicans* cells surviving treatment within 24 h. For comparison, we repeated the experiment with the standard drug amphotericin B, a fungicidal compound (Table 3). Similar to TB_KKG6K, amphotericin B effectively inhibited growth and killed *C. albicans* cells within 24 h. The MFC of amphotericin B was two-fold higher (0.27 μM) than its MIC_90_ (0.14 μM) after 24 h of incubation. At 48 h of incubation, the MIC_90_ doubled to 0.27 µM, while the MFC remained unchanged. These findings underscore the strong and sustained fungicidal potential of TB_KKG6K, comparable to that of amphotericin B, even under conditions that promote biofilm formation.

### 3.2. TB_KKG6K Shows No Adverse Interference with Standard Antifungal Drugs

The employment of combination therapies offers significant advantages, including reducing the risk of pathogenic fungi developing resistance to therapeutic agents and lowering medication dosages. This approach can also help to minimize adverse effects. Therefore, we investigated the interaction between TB_KKG6K and standard drugs, including amphotericin B, caspofungin, fluconazole and 5-FC, in inhibiting fungal growth. The antifungal activity of each drug was first assessed individually using a standard broth microdilution assay to determine their MIC_90_ values (Table 4): TB_KKG6K (2 µM), amphotericin B (0.14 µM), caspofungin (0.11 µM), fluconazole (6.5 µM), and 5-FC (1.9 µM). The drugs were then tested in combination with TB_KKG6K using a checkerboard assay to evaluate their interaction. In combination, TB_KKG6K exhibited an additive effect against *C. albicans* with amphotericin B (FICI = 0.75) and caspofungin (FICI = 0.63). However, it showed an indifferent interaction with fluconazole (FICI = 1.5) or 5-FC (FICI = 2) (Table 4).

To test whether the observed additive effect of TB_KKG6K in combination with amphotericin B or caspofungin influenced the fungicidal activity of the compounds, another checkerboard assay was performed, and the MFC was determined after 24 h and 48 h of incubation. When tested individually, the MFC of the standard drugs was two-fold higher than their MIC_90_ (amphotericin B: 0.27 µM; caspofungin 0.22 µM; Table 4) and remained consistent over the entire incubation period (24 h and 48 h). However, combining the peptide with amphotericin B resulted in a two-fold lower MFC after 24 h of incubation (0.14 µM) compared to amphotericin B alone (0.27 µM). The fungicidal effect was even more pronounced when the peptide was combined with caspofungin, leading to a four-fold lower MFC after 24 h of cultivation (0.055 µM) compared to that of caspofungin alone (0.22 µM). With prolonged incubation, the MFC of the antifungal compound combinations increased; however, in case of amphotericin B, the MFC did not exceed that of the single drug (0.27 µM). For caspofungin, the MFC was 0.11 µM, and remained even below that of the individual compound (0.22 µM).

### 3.3. TB_KKG6K Has Low Potential to Induce Adaptive Mechanisms in C. albicans

The fungicidal mode of action of TB_KKG6K suggests that this remarkable property may minimize the risk of resistance development in *C. albicans*. As proof of principle, we performed an in vitro microevolution experiment to compare the low propensity of *C. albicans* to develop resistance to TB_KKG6K with its tendency to develop resistance to the standard therapeutic agent fluconazole. Fluconazole, a fungistatic drug, is known to elicit adaptive responses in *C. albicans* and can induce resistance mechanisms [32,33,34].

To achieve this aim, *C. albicans* was cultured over successive generations under controlled in vitro conditions with gradually increasing concentrations of TB_KKG6K, starting at 0.5× MIC_90_. Cells treated in the same way with fluconazole served as a positive control for adaptation and resistance induction. An untreated control was included to monitor the growth in the absence of antifungals.

**Figure 1:**
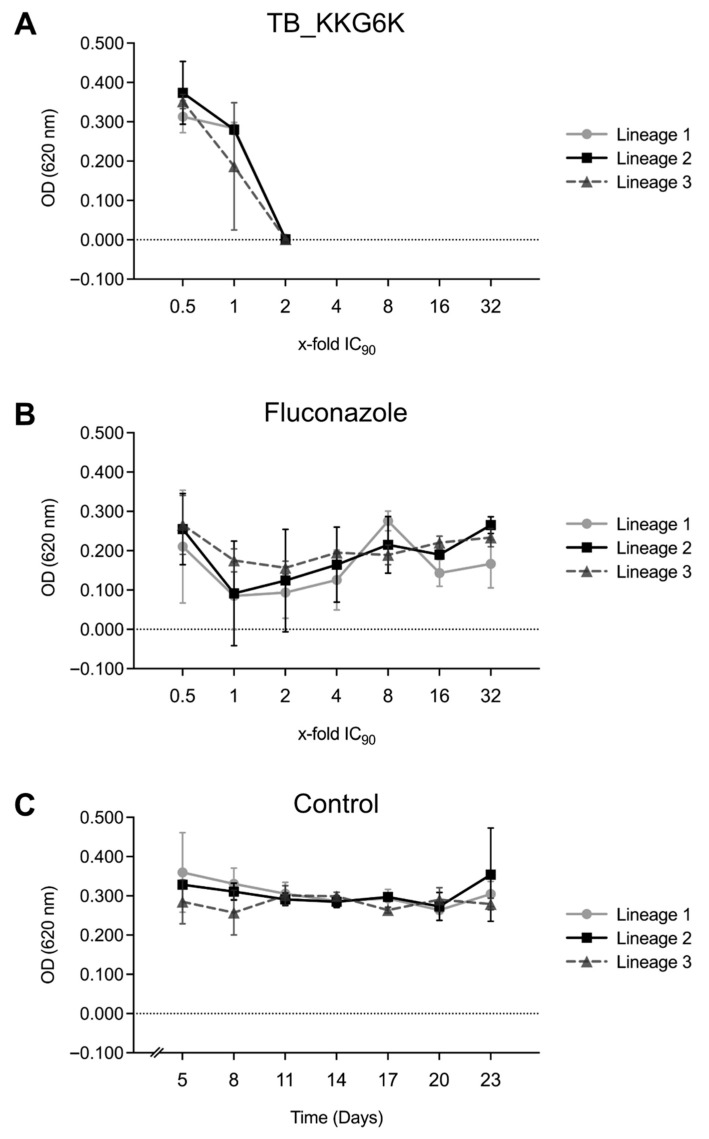
Adaptation of *C. albicans* to increasing concentrations of antifungals. *C. albicans* was exposed to increasing concentrations of (**A**) TB_KKG6K and (**B**) fluconazole, or (**C**) was left untreated (Control). The OD_620_ of three independent lineages, each cultivated in triplicates, were measured at the final passage of the cells in the respective concentrations (in 72-h intervals).

The three lineages of *C. albicans* cells exhibited varying susceptibilities towards 1× MIC_90_ after prolonged exposure (120 h) to the subinhibitory concentration (0.5× MIC_90_) of TB_KKG6K. However, none of the lineages were able to survive serial passages at concentrations exceeding 1× MIC_90_ of TB_KKG6K, as the OD_620_ values dropped to the level of the growth medium used for background control (Figure 1A). This was confirmed by plating aliquots from the third passage of each lineage exposed to 2× MIC_90_ TB_KKG6K on YPD agar plates without antifungal peptide supplementation. No CFU could be counted after the incubation at 30 °C for 24 h. In contrast, cells exposed to gradually increasing concentrations of fluconazole demonstrated the ability to adapt and survive treatment at drug concentration as high as 32× MIC_90_. Initially, all three *C. albicans* lineages showed a decrease in OD_620_ values upon exposure to 1× MIC_90_ of fluconazole. However, a steady recovery was observed over the time, as indicated by progressively increasing OD_620_ values throughout the cultivation period (Figure 1B). Meanwhile, the untreated lineages of the growth control maintained relatively stable OD_620_ values for the entire duration of the experiment (Figure 1C).

### 3.4. TB_KKG6K Inhibits C. albicans Biofilm Development

**Figure 2:**
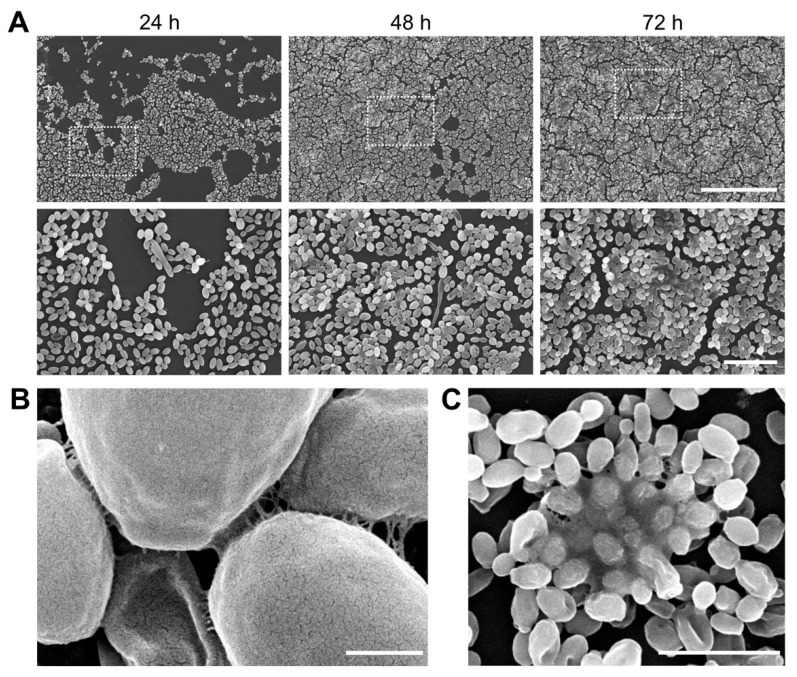
*C. albicans* biofilm and ECM formation. (**A**) Biofilm development on the silicone elastomer discs was recorded in 24-h intervals for 72-h. The images were captured with a JSM-6010LV electron microscope at two different magnifications: 400× (**upper row**), 1400× (**lower row**). Scale bars 100 µm and 20 µm, respectively. The dotted square in the images of the upper row indicate the magnified section shown in the lower row. (**B**) A detailed image of ECM formation in a 48-h old biofilm taken with a TESCAN CLARA electron microscope. Scale bar, 1 µm. (**C**) Dense accumulation of ECM in a 72-h old biofilm captured with a JSM-6010LV SEM. Scale bar, 10 µm.

To study the efficacy of TB_KKG6K on sessile *C. albicans* growing on synthetic material, we selected silicone elastomer to first establish biofilm maturation. Silicone elastomer discs were inoculated with *C. albicans*, after which biofilm development was observed at 24-h intervals over a 72-h period using SEM. Following 24 h of incubation, the discs exhibited a moderate degree of biofilm density, comprising predominantly spherical yeast cells (Figure 2A). A 48-h biofilm exhibited higher cell density, manifesting in the formation of cell clusters as described previously [35,36]. At this stage of biofilm development, some germinating cells and pseudohyphae, and the formation of ECM between the cells could be observed (Figure 2A,B). Cell density and clustering increased progressively over the next 24 h, ultimately forming a multilayered biofilm by 72 h (Figure 2A) with ECM dense areas (Figure 2C).

To study the efficacy of TB_KKG6K in inhibiting biofilm maturation on silicone, it was necessary to determine the appropriate concentration, sufficiently high to affect the cells growing in a biofilm, yet low enough to preserve adequate cell material for analysis. To achieve this, the reduction in viable sessile *C. albicans* cells embedded in a 48-h old biofilm was quantified using a CFU assay after exposure to TB_KKG6K for a short (4 h) and long (24 h) incubation period. Results were compared to a control biofilm that remained unexposed to the peptide for the same incubation times. The number of viable cells was decreased compared to the untreated control cells 4 h after the administration of TB_KKG6K at 1× MIC_90_ (2 µM), though this change did not reach statistical significance (Table 5). However, the application of TB_KKG6K at higher concentrations (≥5 µM) demonstrated a concentration-dependent reduced number of viable cells compared to the untreated control, which was statistically significant (Table 5). Notably, treatment with 50 µM TB_KKG6K reduced CFUs to 0.8%, comparable to amphotericin B treatment (CFU reduction to 0.1%) relative to the untreated control (Table 5).

After 24 h of exposure, peptide concentrations close to the MIC_90_/MFC (2–5 µM) did not lead to a significant decrease in CFUs compared to the untreated control. Interestingly, there was a slight increase in CFU at 2 µM, though this change was not statistically significant. However, at higher peptide concentrations (≥10 µM), a marked decline in CFUs was observed, with the number of viable cells reduced to 48.9% relative to the untreated control. At 50 µM TB_KKG6K, this effect was further amplified, resulting in only 4.5% viable cells in the biofilm compared to the untreated control (Table 5). The treatment with 1.4 µM amphotericin B resulted in a CFU reduction to 0.6% (Table 5).

**Figure 3:**
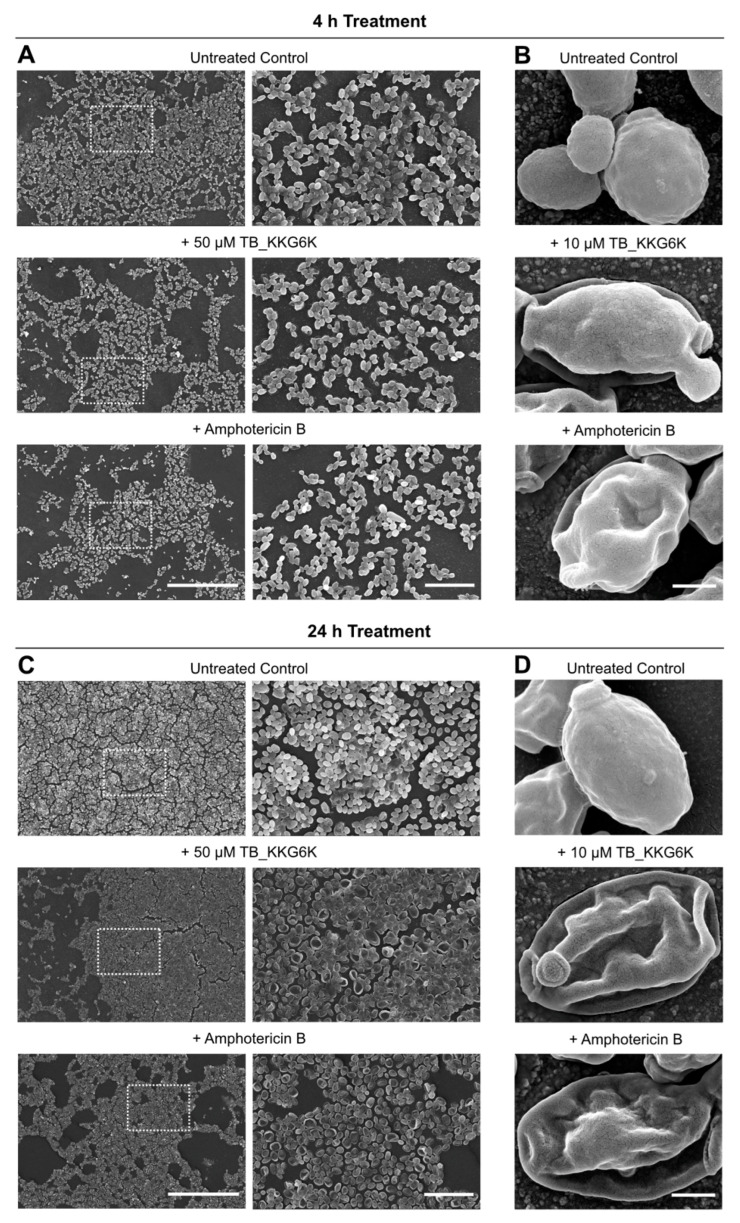
The effect of TB_KKG6K on *C. albicans* cells growing in a biofilm. A 48-h old biofilm was treated with TB_KKG6K (50 µM) or amphotericin B (1.4 µM) for 4 h (**A**) or 24 h (**C**) and compared to the untreated control. The images were captured with a JSM-6010LV electron microscope at two different magnifications: 400× (**left**), 1400× (**right**). Scale bars 100 µm and 20 µm, respectively. The dotted square in the **left** panels indicate the magnified section shown in the **right** image. Detailed image of cells grown in a 48-h old biofilm and treated with 10 µM TB_KKG6K or 1.4 µM amphotericin B for 4 h (**B**) or 24 h (**D**). Untreated cells served as control. Images were taken with a TESCAN CLARA SEM. Scale bar, 1 µm.

The impact of a 4-h and 24-h exposure to 50 µM TB_KKG6K (25× MIC_90_) on a matured biofilm was visualized using SEM. After 4 h of treatment, cell clustering was reduced, while severe cell damage was observed following 24-h treatment compared to the untreated biofilm (Figure 3A,C). Similar effects were noted when the biofilm was treated with 1.4 µM of amphotericin B (10× MIC_90_; Figure 3A–D). These effects were absent in the untreated control (Figure 3B,D). To further capture the progression of cell damage caused by TB_KKG6K, the 48-h old biofilm was also exposed to a lower peptide concentration (10 µM), corresponding to 5× MIC_90_, for the same incubation times (4 h and 24 h). Under these conditions, only a few cells with irregular yeast morphology could be observed after 4 h (Figure 3B). The number of these cells exhibiting completely collapsed structure significantly increased after 24 h of incubation (Figure 3D).

### 3.5. TB_KKG6K Reduces ECM Formation in C. albicans Biofilm

**Figure 4:**
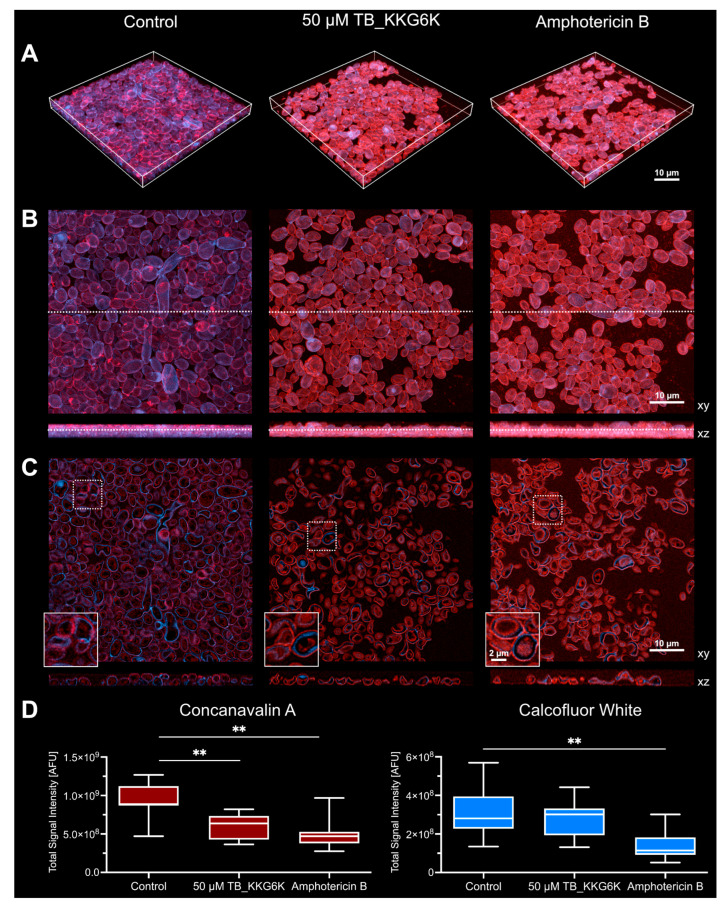
Effect of TB_KKG6K treatment on *C. albicans* biofilm structure. Biofilm that had matured for 48 h was treated with 50 µM TB_KKG6K or 1.4 µM amphotericin B for 24 h. The fixed samples were stained with 100 µg mL^−1^ Concanavalin A-AF633 (red) and 1 mg mL^−1^ Calcofluor White (blue). Samples were examined using a SP8 gSTED microscope at a magnification of 93×. (**A**) Images display the maximum intensity profile (MIP) projection in three dimensions (3D). (**B**) Images present the MIP in two dimensions (2D), with the upper panel oriented in the xy and the lower panel oriented in the xz direction. The dotted lines delineate the region from which sections were selected for depiction in (**C**). (**C**) Images show the MIP in 2D, with the upper panel in xy and the lower panel in xz orientation. The dotted squares denote the specific regions of xy that were enlarged in the images displayed in the lower left corner. (**D**) The total signal quantifications per image was measured for the entire z stack with the Huygens Professional software 25.04 and is expressed in arbitrary fluorescence units (AFU). Three representative recordings of each experimental condition from the biological replicates (n = 3) were used, and the resulting data points were arranged in box plots. (** *p* ≤ 0.005).

We next analyzed the structural composition of the biofilm using confocal microscopy with the fluorescent stains Calcofluor White and Concanavalin A-AF633 to visualize the cell wall of *Candida* cells and the ECM. In the untreated control, the biofilm exhibited a dense structure predominantly composed of round and oval-shaped yeast cells, including budding, and some germinating and pseudohyphae (Figure 4A–C). The majority of cells were embedded in ECM, as evidenced by Concanavalin A-AF633 signal, and displayed ECM accumulation in specific regions of the cell surface, reflecting the characteristic heterogeneity of biofilms [7,37]. The fungal cells were counterstained with Calcofluor White, which binds cell wall chitin. Accumulation of Concanvalin A and Calcofluor White signals at specific sites of the cells coincided also with bud scars [38]. Higher magnification imaging revealed that ECM surrounded the cell wall of *C. albicans*. Treatment with TB_KKG6K resulted in a noticeable reduction in the cell density, accompanied by the presence of collapsed cells (Figure 4C), an effect that paralleled the observations made with amphotericin B treatment and the findings from SEM analysis (Figure 3). Quantification of the total signal intensities of Concanavalin-AF633 revealed a significant reduction in the glucan component of the ECM and cell wall in the TB_KKG6K-treated biofilms compared to the untreated control, similarly to the effect observed with amphotericin B (Figure 4D). The Calcofluor White signal, which reflects chitin content, was reduced in the TB_KKG6K-treated biofilms, although the reduction was less pronounced than in the amphotericin B-treated samples, but still notable compared to the untreated control (Figure 4D).

### 3.6. TB_KKG6K Induces Transcriptional Deregulation in Sessile C. albicans Cells

**Figure 5:**
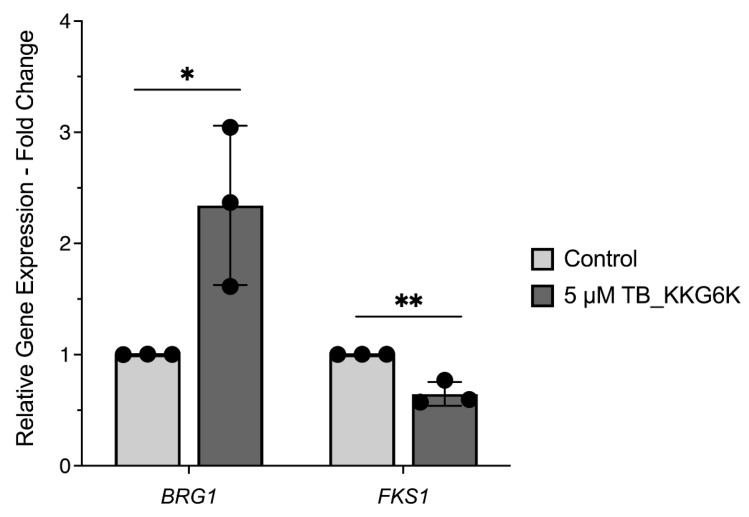
Relative gene expression analysis by RT-qPCR. *C. albicans* biofilm that had matured for 48 h was treated for 4 h with 5 µM TB_KKG6K. Gene expression levels were determined using the ΔΔ Ct method. Expression of the target genes *BRG1* and *FKS1* was normalized to the geometric mean of the two housekeeping genes *ACT1* and *EFB1* and is presented as fold change relative to the untreated control. Bars represent mean ± SD from three independent biological replicates (n = 3). Statistical significance was determined by Student’s *t*-test. (* *p* ≤ 0.05 and ** *p* ≤ 0.005).

To evaluate the nuclear response of *C. albicans* cells growing in a matured biofilm to TB_KKG6K exposure, RT-qPCR was performed. To circumvent global secondary effects induced in dying cells and ensure the recovery of sufficient quantity of material, we applied a lower peptide concentration (5 µM TB_KKG6K) and a short incubation time (4-h treatment of a 48-h old biofilm). We investigated the gene transcription of the general biofilm regulating transcription factor *BRG1* and the 1,3-β-D-glucan synthase catalytic subunit *FKS1* using the expression of the house-keeping genes coding for actin (*ACT1*) and elongation factor 1-beta (*EFB1*), as previously described [39]. RT-qPCR revealed that TB_KKG6K induced a 2.34-fold increase in the expression of *BRG1* and a 0.64-fold decrease in *FKS1* gene expression (Figure 5).

## 4. Discussion

The potential of new antifungal compounds to drive the development of novel drugs relies strongly on preclinical in vitro studies that investigate their mode of action and antimicrobial efficacy. These investigations typically evaluate key factors, including fungicidal or fungistatic activity, anti-biofilm properties, the potential to induce resistance in the pathogen, efficacy in combination with standard drugs, and tolerability in host cells. Previous research has shown that the amphibian-derived temporin B analog, TB_KKG6K, rapidly kills planktonic *C. albicans* cells as well as those growing in biofilms, while being well tolerated in human cells [20,21,22]. The present proof-of-concept study expands our knowledge on its fungicidal potential and, for the first time, provides evidence that TB_KKG6K can be combined with standard drugs without affecting their fungicidal efficacy against *C. albicans*, and has a low potential to induce resistance in this opportunistic human pathogenic yeast. Furthermore, it demonstrates that this peptide inhibits *C. albicans* biofilm maturation on medically relevant material.

Susceptibility testing using broth microdilution assays revealed that TB_KKG6K inhibited *C. albicans* growth at a low μM concentration as reported previously [20], exhibiting a MIC_90_ in a similar range as that of 5-FC and fluconazole, but higher than amphotericin B and caspofungin which were effective at nM concentrations. The combination of the peptide with these standard drugs in a checkerboard assay demonstrated that TB_KKG6K had no synergistic or antagonistic effects on *C. albicans* growth. While this excludes a potentiation of antifungal activity, it also ensures that the peptide does not negatively interfere with existing treatments. Instead, its combination with amphotericin B or caspofungin demonstrated an additive interaction (FICI 0.75 or 0.63, respectively) exhibiting fungicidal activity. This result could be clinically significant, as a combination may help mitigate resistance development in the fungal pathogen, reduce dosages, and improve drug tolerability in the patient.

Notably, *C. albicans* showed only a mild adaptation to 0.5–1× MIC_90_ of TB_KKG6K under prolonged cultivation but was readily killed when transferred to medium supplemented with the peptide at concentrations exceeding 1× MIC_90_. This suggests that the peptide has low potential to induce resistance mechanisms in planktonic yeast cells. The result aligns well with our recently published studies, describing the fast fungicidal activity of TB_KKG6K in *C. albicans* and a complex, potentially multifaceted mode of action, which is associated with cell membrane activity, entry into the yeast cell, iROS generation, and disintegration of intracellular membranes and organelles [20,21]. Moreover, TB_KKG6K also inhibited the growth of a fluconazole-resistant *C. albicans* species, which underscore its robust antifungal efficacy [20]. In contrast to TB_KKG6K, *C. albicans* was able to survive prolonged subcultivation in the presence of increasing fluconazole concentrations up to 32× MIC_90_. Thus, the TB_KKG6K acts differently to fluconazole, which is known to be fungistatic and has a high tendency to induce multiple resistance mechanisms in *C. albicans* [32,33,34], including the upregulation of drug efflux transporters, mutations in *ERG11*, and overexpression of lanosterol 14-α-demethylase [40,41,42]. Some AMPs with antifungal activity have been shown to induce tolerance in *C. albicans* upon sequential exposure to increasing peptide concentrations, e.g., fungal NFAP2 and human salivary histatin 3. However, adaptation mechanisms that resulted in tolerance remained unsolved [26,43]. Future studies are needed to determine whether the findings obtained with planktonic cells of one *C. albicans* strain tested in vitro can be translated to drug-resistant strains or non-*albicans Candida* species, as well as to in vivo conditions, particularly for cells growing within a biofilm.

Biofilms are defined as complex-surface-associated microbial communities embedded in an ECM composed of glycoproteins, polysaccharides, lipids and nucleic acids. Compared to their planktonic counterparts, biofilm-associated cells show altered gene expression and growth, and are benefitting from ECM mediated protection against environmental stress, immune responses, and antimicrobial agents [44,45,46]. *C. albicans* biofilms represent a major challenge in the treatment of fungal infections due to their inherent resistance to antifungal drugs. This is particularly problematic, where persistent biofilms lead to severe infections and contamination of medical devices. In one of our previous studies, we could demonstrate that TB_KKG6K exhibited growth-inhibitory efficacy against 24-h old sessile *C. albicans* cells. However, these biofilms were cultivated on conventional laboratory plastic rather than clinically relevant materials [20]. Therefore, the objective of this study was to characterize in greater detail the activity of TB_KKG6K against matured *C. albicans* biofilms formed on material with clinical relevance.

We selected silicone elastomer, which has found practical application in many biomedical devices, including the tubing of urinary and peritoneal catheters, wound dressing, shunts and drains, contact lenses, orthopedics and many more [47]. Silicone material has been used in studies addressing device-associated *C. albicans* infections [48,49,50] and natural product-based treatments [51,52].

TB_KKG6K demonstrated strong efficacy against matured 48-h old *C. albicans* biofilm on silicone, with a time and concentration dependent reduction in CFU counts determined after treatment within a micromolar peptide concentration range. A statistically significant reduction in biofilm-associated cells, quantified as CFU numbers, was reached after 24 h in the presence of 25× MIC_90_ of TB_KKG6K. A similar effect was observed with the drug control sample exposed to 10× MIC_90_ amphotericin B. Notably, the decrease in CFU numbers of sessile cells exposed to the peptide for only 4 h was relatively higher when compared to a 24-h treatment. This indicates that cells growing in a biofilm are more vulnerable to the antifungal during a short time exposure than a prolonged exposure. This phenomenon may be explained by a decrease in antifungal efficacy over time. The peptide could be degraded by proteases secreted by the yeast cells, and/or few cells that survived the treatment could resume growth, as quantified by the CFU numbers. These proliferating cells could be a source of secreted protective ECM material. However, technical issues could also explain the above-described phenomenon. Although carefully performed, multiple pipetting steps applied in the preparation of samples for imaging might destabilize cells growing in a biofilm, causing them to detach from the surface and be discharged with the supernatant when it is aspirated off. Therefore, the results obtained through imaging cannot be fully aligned with those obtained through CFU assays. Since antifungal activity in biofilms is both time and concentration dependent, a significant challenge arises in determining whether the drug can be effectively delivered to the site of infection and whether fungicidal concentrations can realistically be achieved and maintained over time. To address this, effective antifungal treatment strategies in the clinics involve the repeated application of a drug or drug combinations to ensure the eradication of the infectious agent.

For analyzing the biofilm structure, the application of SEM was particularly informative in regions of high cell density and ECM accumulation, providing robust evidence for the disruptive potential of the tested peptide against *C. albicans* biofilm integrity. It demonstrated the collapse of *C. albicans* cells grown within the biofilm after treatment with the peptide. Similarly collapsed cells could be observed in the amphotericin B treated control, which was consistent with other studies that showed similar cell deformation to occur in response to drug exposure [53,54]. No signs of cell wall roughening, pores or other cell surface damages were visible after TB_KKG6K treatment. This aligns with our previous observation obtained with transmission electron microscopy, which revealed the disruption of intracellular membrane structures in planktonic *C. albicans* cells upon peptide treatment, but without pore formation in the cell membrane as an initial trigger for cell death [21].

By using confocal microscopy, we visualized components of the cell wall and the ECM of *C. albicans* cells growing within a biofilm. The cell wall of *C. albicans* exhibits a complex structure, consisting of multiple components organized into two distinct layers. The cell wall’s primary structure is a chitin-β-glucan-mannoprotein framework, wherein chitin is located in the inner layer and glucans together with mannans in the outer layer of the cell wall [55,56]. Our observations made with confocal microscopy aligned with this structure description (Figure 4). The fluorescent dye Calcofluor White exhibits a high affinity for chitin and cellulose [57]. We observed a mild reduction in Calcofluor White signal intensity in the TB_KKG6K-treated biofilm, while the decrease in signal intensity was more pronounced in biofilms treated with amphotericin B. This might be explained by the ability of this drug to extract ergosterol from the membrane, thereby inducing stress response and impairing the cell membrane function, ultimately compromising the integrity of the inner cell wall [58,59,60,61,62].

The ECM produced by *C. albicans* contains mannans, β-1,6- and β-1,3-glucans, which assemble extracellularly to form a structure that sequesters antifungal agents, blocking their access to cellular targets [63,64,65]. Concanavalin A, a plant lectin that binds to α-mannopyranosyl and α-glucopyranosyl residues, is used to stain the outer layer of the fungal cell walls and the ECM, when fluorochrom-conjugated [66]. TB_KKG6K, similar to amphotericin B, led to a significant reduction in the Concanavalin A-AF633 signal intensities compared to the untreated controls. This observation can be explained by the reduction in viable cells, documented by a decrease in CFU, and the reduction in cell wall/ECM components through TB_KKG6K. The latter hypothesis is supported by identifying a significant reduction in the expression of the *FKS1* gene encoding the catalytic subunit of the β-1,3-glucan synthase complex with RT-qPCR. Notably, the gene transcription data were collected after a 4-h long peptide exposure of biofilm, while changes in the cell wall and ECM composition were visualized microscopically after a 24-h long incubation. However, downregulation of *FKS1* by TB_KKG6K treatment may alter in the long-term cell wall composition and compromise the protective function of ECM, enhancing biofilm disruption. The cell wall and ECM component β-1,3-glucan plays a critical role in the virulence of *C. albicans*. Strains with impaired Fks1 function show reduced fitness, and are less able to undergo yeast-to-hyphae transition and to establish infection in vivo [67]. The enzymatic targeting of the ECM with β-1,3-glucanases was shown to increase drug susceptibility and facilitate biofilm eradication [68,69]. However, based on our previous observation that TB_KKG6K does not localize to the nucleus of the yeast cell [21], we hypothesize that deregulation of gene expression occurs in response to changes in signal transduction, as it has been reported to occur also with other antifungal compounds [70,71].

*C. albicans* biofilms exhibit a tightly regulated morphogenetic plasticity, characterized by transition between yeast and hyphal forms. A complex signaling network, including master transcription factors such as Brg1, influence the bi-directional transition. These factors control the expression of a coordinated gene network that is essential for dynamic morphological remodeling and biofilm structure [72,73,74]. SEM and confocal microscopy revealed that *C. albicans* primarily formed a biofilm composed of yeast cells containing only occasionally germinating cells or pseudohyphae, and this observation did not change upon treatment with antifungals. Nevertheless, *BRG1* was found to be overexpressed in the biofilm established on silicone in response to a 4-h TB_KKG6K treatment. We propose that upregulation of *BRG1* could be a compensatory stress response of *C. albicans* aimed at restoring biofilm integrity. Stress-mediated modulation of Tor1 signaling in yeast cells has been reported to be an important factor in the regulation of *BRG1* [30,75,76]. This hypothesis certainly merits further analysis in the future.

We have previously been able to show the excellent biocompatibility of TB_KKG6K when applied to keratinocyte-based reconstructed 3D skin models in two independent studies [20,22]. Its fungicidal mode of action, low risk of resistance development and demonstrated anti-biofilm efficacy, as evidenced in the present study, underscore the significant potential of this peptide for managing chronic or recurrent *C. albicans* infections and preventing colonization of abiotic surfaces. In addition, TB_KKG6K exhibited no antagonistic interactions with standard antifungal drugs, enabling its use in combination therapies at reduced dosages. Notably, TB_KKG6K may be especially suitable for use in catheter-associated infections, as it is expected to be compatible with silicone materials when delivered in water-based formulations. This makes it a promising candidate for antifungal lock therapy, a treatment approach that involves instilling a high-concentration antifungal agent into colonized intravascular catheters to achieve sterilization, particularly when catheter removal is not feasible and systemic therapy alone is insufficient [77,78]. An appropriate formulation for the repeated application of TB_KKG6K may be a crucial step to enhance efficacy in anti-*Candida* treatment. Innovative drug delivery approaches offer promising solutions to tackle these challenges, enhancing biofilm penetration and ensuring targeted peptide delivery at the infection site [79]. Furthermore, formulated TB_KKG6K could have great potential to be used in catheter coatings and wound dressings to impede colonization and infection with *C. albicans*, respectively. A feasible example was given with temporin SHa, a member of the temporin family that was successfully grafted onto gold surfaces, demonstrating effective surface functionalization [80,81].

In this study, we utilized a silicone elastomer model that provides a controlled environment for studying biofilm formation and evaluating antifungal treatment on silicone-based devices. However, this model does not account for in vivo factors such as host interactions and immune response, and its clinical relevance remains to be determined. Despite these limitations, the study establishes a foundation for future research on treating biofilms on medically relevant materials with natural products like TB_KKG6K.

## Figures and Tables

**Table 1:** Media and solutions used in this study.

Media/Solution	Composition ^$^
Potato Dextrose Broth (PDB)	0.65% Potato infusion, 2% Glucose
Potato Dextrose Agar (PDA)	Potato Dextrose Broth, 2% Agar
Phosphate-Buffered Saline (PBS)	0.05% KH_2_PO_4_, 0.28% K_2_HPO_4_, 0.9% NaCl, pH 7.4
Dulbecco’s phosphate-buffered saline (D-PBS)	0.02% KCl, 0.02% KH_2_PO_4_, 0.8% NaCl, 0.115% Na_2_HPO_4_, pH 7.0
Yeast Peptone D-Glucose (YPD) Agar	1% Yeast extract, 2% Peptone, 2% Glucose, 2% Agar

^$^ Percent values (%) are depicted as (wt/vol).

**Table 2:** Primers used in this study.

Gene	Orientation	Sequence 5′–3′	Source
*ACT1*	Forward	GCTGGTAGAGACTTGACCAACCA	[28]
	Reverse	GACAATTTCTCTTTCAGCACTAGTAGTGA	
*EFB1*	Forward	CAGCCGCTTCTGGTTCTGCT	This study
	Reverse	AGCAGCCTTCTTAGCAGCGT	
*BRG1*	Forward	AGCTGGTGTGCCACCTCCAC	[30]
	Reverse	TACCACACCTGTGACATCTG	
*FKS1*	Forward	GGATATCAAGACCAAGCCAACTA	[31]
	Reverse	CCAGGAGTTTGACCACCATAA	

**Table 3:** MIC_90_ and MFC of tested compounds.

	MIC90 [µM]		MFC [µM]	
Compound	24 h	48 h	24 h	48 h
TB_KKG6K	2	2	2	2
Amphotericin B	0.14	0.27	0.27	0.27

**Table 4:** FICI of licensed drugs and TB_KKG6K against planktonic *C. albicans* cells.

		Median (Range)			
Agent	MIC_90_ [µM]	FIC_A_ [TB_KKG6K]	FIC_B_ [Licensed Antifungal]	FICI [FIC_A_ + FIC_B_]	Interpretation
TB_KKG6K	2	-	-	-	-
Amphotericin B	0.14	0.5 (0.25–0.5)	0.5 (0.25–0.5)	0.75 (0.75–1)	Additive
Caspofungin	0.11	0.5 (0.25–0.5)	0.13 (0.06–0.5)	0.63 (0.56–0.75)	Additive
Fluconazole	6.5	0.5 (0.5–1)	0.5 (0.5–1)	1.5 (1–1.5)	Indifferent
5-FC	1.9	1	1	2	Indifferent

**Table 5:** Survival of sessile *C. albicans* cells grown on silicone and treated with increasing concentrations of TB_KKG6K for 4 h or 24 h ^§^.

		4 h Treatment			24 h Treatment		
		CFU cm^−2^	log_10_ Difference ^&^	Survival [%] ^#^	CFU cm^−2^	log_10_ Difference ^&^	Survival [%] ^#^
Control		(1.85 ± 0.56) × 10^6^	0	100	(3.42 ± 0.86) × 10^6^	0	100
TB_KKG6K	2 µM	(1.73 ± 0.52) × 10^6^	−0.03 ± 0.07	95.4 ± 13.8	(4.75 ± 1.72) × 10^6^	+0.13 ± 0.05	134.9 ± 16.2
	5 µM	(8.87 ± 0.74) × 10^5^ *	−0.30 ± 0.13	52.9 ± 16.7	(3.36 ± 1.55) × 10^6^	−0.06 ± 0.18	93.0 ± 33.8
	10 µM	(3.34 ± 2.58) × 10^5^ **	−0.92 ± 0.35	16.0 ± 11.5	(1.56 ± 0.09) × 10^6^	−0.33 ± 0.12	48.9 ± 14.4
	50 µM	(1.14 ± 0.37) × 10^4^ **	−2.21 ± 0.28	0.8 ± 0.5	(1.21 ± 0.97) × 10^5^ *	−1.58 ± 0.45	4.5 ± 4.6
Amphotericin B		(2.39 ± 0.94) × 10^3^ **	−2.90 ± 0.10	0.1 ± 0.0	(2.22 ± 0.66) × 10^4^ *	−2.20 ± 0.06	0.6 ± 0.1

^§^ All values are presented as mean ± standard deviation. Silicone discs were inoculated with 5 × 10^5^ cells and incubated for 48 h. Biofilm that had matured for 48 h underwent antifungal treatment (2–50 µM TB_KKG6K; 1.4 µM amphotericin B) for 4 h or 24 h. The cells were harvested and plated in serial dilutions on PDA. CFU were quantified after 24 h of incubation. The absolute CFU counts cm^−2^ silicone disc are given for three biological replicates (n = 3) each conducted in duplicates. (* *p* ≤ 0.05; ** *p* ≤ 0.005). ^&^ The absolute CFU data were log_10_ transformed. The log_10_ difference was determined based on the comparison of the treated with the untreated biofilm control within each biological replicate [log_10_(CFU_control_) − log_10_(CFU_Treatment_)]. ^#^ The relative survival (%) represents the average percentage of viable CFUs of treated biofilm compared to the untreated biofilm control at the time of harvest and was calculated from absolute CFU data within each biological replicate as [(CFU_Treatment_/CFU_Control_) × 100].

## Data Availability

The original contributions presented in this study are included in the article. Further inquiries can be directed to the corresponding authors.

## References

[1] Nobile C.J., Johnson A.D. (2015). *Candida albicans* Biofilms and Human Disease. Annu. Rev. Microbiol..

[2] Gulati M., Nobile C.J. (2016). *Candida albicans* biofilms: Development, regulation, and molecular mechanisms. Microbes Infect..

[3] Atriwal T., Azeem K., Husain F.M., Hussain A., Khan M.N., Alajmi M.F., Abid M. (2021). Mechanistic Understanding of *Candida albicans* Biofilm Formation and Approaches for Its Inhibition. Front. Microbiol..

[4] Thomas-Rüddel D.O., Schlattmann P., Pletz M., Kurzai O., Bloos F. (2022). Risk Factors for Invasive *Candida* Infection in Critically Ill Patients: A Systematic Review and Meta-analysis. Chest.

[5] Lass-Flörl C., Kanj S.S., Govender N.P., Thompson G.R., Ostrosky-Zeichner L., Govrins M.A. (2024). Invasive candidiasis. Nat. Rev. Dis. Primers.

[6] Finkel J.S., Mitchell A.P. (2011). Genetic control of *Candida albicans* biofilm development. Nat. Rev. Microbiol..

[7] Chandra J., Kuhn D.M., Mukherjee P.K., Hoyer L.L., McCormick T., Ghannoum M.A. (2001). Biofilm formation by the fungal pathogen *Candida albicans*: Development, architecture, and drug resistance. J. Bacteriol..

[8] Nett J., Andes D. (2006). *Candida albicans* biofilm development, modeling a host-pathogen interaction. Curr. Opin. Microbiol..

[9] Malinovská Z., Čonková E., Váczi P. (2023). Biofilm Formation in Medically Important *Candida* Species. J. Fungi.

[10] d’Enfert C. (2006). Biofilms and their role in the resistance of pathogenic *Candida* to antifungal agents. Curr. Drug Targets.

[11] Ramage G., Martínez J.P., López-Ribot J.L. (2006). *Candida* biofilms on implanted biomaterials: A clinically significant problem. FEMS Yeast Res..

[12] Talpaert M.J., Balfour A., Stevens S., Baker M., Muhlschlegel F.A., Gourlay C.W. (2015). *Candida* biofilm formation on voice prostheses. J. Med. Microbiol..

[13] Bouza E., Guinea J., Guembe M. (2014). The Role of Antifungals against *Candida* Biofilm in Catheter-Related Candidemia. Antibiotics.

[14] Ponde N.O., Lortal L., Ramage G., Naglik J.R., Richardson J.P. (2021). *Candida albicans* biofilms and polymicrobial interactions. Crit. Rev. Microbiol..

[15] Mateus C., Crow S.A., Ahearn D.G. (2004). Adherence of *Candida albicans* to silicone induces immediate enhanced tolerance to fluconazole. Antimicrob. Agents Chemother..

[16] Mukherjee P.K., Chandra J., Kuhn D.M., Ghannoum M.A. (2003). Mechanism of fluconazole resistance in *Candida albicans* biofilms: Phase-specific role of efflux pumps and membrane sterols. Infect. Immun..

[17] Ramage G., Bachmann S., Patterson T.F., Wickes B.L., López-Ribot J.L. (2002). Investigation of multidrug efflux pumps in relation to fluconazole resistance in *Candida albicans* biofilms. J. Antimicrob. Chemother..

[18] D’Andrea L.D., Romanelli A. (2023). Temporins: Multifunctional Peptides from Frog Skin. Int. J. Mol. Sci..

[19] Avitabile C., D’Andrea L.D., D’Aversa E., Milani R., Gambari R., Romanelli A. (2018). Effect of Acylation on the Antimicrobial Activity of Temporin B Analogues. ChemMedChem.

[20] Kakar A., Holzknecht J., Dubrac S., Gelmi M.L., Romanelli A., Marx F. (2021). New Perspectives in the Antimicrobial Activity of the Amphibian Temporin B: Peptide Analogs Are Effective Inhibitors of *Candida albicans* Growth. J. Fungi.

[21] Kakar A., Sastré-Velásquez L.E., Hess M., Galgóczy L., Papp C., Holzknecht J., Romanelli A., Váradi G., Malanovic N., Marx F. (2022). The Membrane Activity of the Amphibian Temporin B Peptide Analog TB_KKG6K Sheds Light on the Mechanism That Kills *Candida albicans*. mSphere.

[22] Schöpf C., Knapp M., Scheler J., Coraça-Huber D.C., Romanelli A., Ladurner P., Seybold A.C., Binder U., Würzner R., Marx F. (2025). The antibacterial activity and therapeutic potential of the amphibian-derived peptide TB_KKG6K. mSphere.

[23] EUCAST (2023). EUCAST Definitive Document, E.Def 7.4: Method for the Determination of Broth Dilution Minimum Inhibitory Concentrations of Antifungal Agents for Yeasts. https://www.eucast.org/fungi-afst/methodology-and-instructions/ast-of-yeasts/.

[24] Meletiadis J., Verweij P.E., TeDorsthorst D.T., Meis J.F., Mouton J.W. (2005). Assessing in vitro combinations of antifungal drugs against yeasts and filamentous fungi: Comparison of different drug interaction models. Med. Mycol..

[25] Kovács R., Nagy F., Tóth Z., Forgács L., Tóth L., Váradi G., Tóth G.K., Vadászi K., Borman A.M., Majoros L. (2021). The *Neosartorya fischeri* Antifungal Protein 2 (NFAP2): A New Potential Weapon against Multidrug-Resistant *Candida auris* Biofilms. Int. J. Mol. Sci..

[26] Bende G., Zsindely N., Laczi K., Kristóffy Z., Papp C., Farkas A., Tóth L., Sáringer S., Bodai L., Rákhely G. (2025). The *Neosartorya (Aspergillus) fischeri* antifungal protein NFAP2 has low potential to trigger resistance development in *Candida albicans* in vitro. Microbiol. Spectr..

[27] Geschwindt A. (2023). Studies on the Antifungal Potential of Small, Cationic, Peptides Against *Candida albicans*. Master’s Thesis.

[28] Oliveira V.C., Souza M.T., Zanotto E.D., Watanabe E., Coraça-Huber D. (2020). Biofilm Formation and Expression of Virulence Genes of Microorganisms Grown in Contact with a New Bioactive Glass. Pathogens.

[29] Livak K.J., Schmittgen T.D. (2001). Analysis of relative gene expression data using real-time quantitative PCR and the 2^−ΔΔCT^ Method. Methods.

[30] Lu Y., Su C., Liu H. (2012). A GATA transcription factor recruits Hda1 in response to reduced Tor1 signaling to establish a hyphal chromatin state in *Candida albicans*. PLoS Pathog..

[31] Sah S.K., Yadav A., Kruppa M.D., Rustchenko E. (2023). Identification of 10 genes on *Candida albicans* chromosome 5 that control surface exposure of the immunogenic cell wall epitope β-glucan and cell wall remodeling in caspofungin-adapted mutants. Microbiol. Spectr..

[32] Sun L.L., Li H., Yan T.H., Fang T., Wu H., Cao Y.B., Lu H., Jiang Y.Y., Yang F. (2023). Aneuploidy Mediates Rapid Adaptation to a Subinhibitory Amount of Fluconazole in *Candida albicans*. Microbiol. Spectr..

[33] Gerstein A.C., Berman J. (2020). Genetic Background Influences Mean and Heterogeneity of Drug Responses and Genome Stability during Evolution in Fluconazole. mSphere.

[34] Morschhäuser J. (2016). The development of fluconazole resistance in *Candida albicans*—An example of microevolution of a fungal pathogen. J. Microbiol..

[35] McCall A.D., Pathirana R.U., Prabhakar A., Cullen P.J., Edgerton M. (2021). Author Correction: *Candida albicans* biofilm development is governed by cooperative attachment and adhesion maintenance proteins. NPJ Biofilms Microbiomes.

[36] Wesenberg-Ward K.E., Tyler B.J., Sears J.T. (2005). Adhesion and biofilm formation of *Candida albicans* on native and Pluronic-treated polystyrene. Biofilms.

[37] Bonhomme J., d’Enfert C. (2013). *Candida albicans* biofilms: Building a heterogeneous, drug-tolerant environment. Curr. Opin. Microbiol..

[38] de Assis L.J., Bain J.M., Liddle C., Leaves I., Hacker C., Peres da Silva R., Yuecel R., Bebes A., Stead D., Childers D.S. (2022). Nature of β-1,3-Glucan-Exposing Features on *Candida albicans* Cell Wall and Their Modulation. mBio.

[39] Hebecker B., Vlaic S., Conrad T., Bauer M., Brunke S., Kapitan M., Linde J., Hube B., Jacobsen I.D. (2016). Dual-species transcriptional profiling during systemic candidiasis reveals organ-specific host-pathogen interactions. Sci. Rep..

[40] Whaley S.G., Berkow E.L., Rybak J.M., Nishimoto A.T., Barker K.S., Rogers P.D. (2016). Azole Antifungal Resistance in *Candida albicans* and Emerging Non-*albicans Candida* Species. Front. Microbiol..

[41] Selmecki A., Forche A., Berman J. (2006). Aneuploidy and isochromosome formation in drug-resistant *Candida albicans*. Science.

[42] Pristov K.E., Ghannoum M.A. (2019). Resistance of *Candida* to azoles and echinocandins worldwide. Clin. Microbiol. Infect..

[43] Fitzgerald D.H., Coleman D.C., O’Connell B.C. (2003). Binding, internalisation and degradation of histatin 3 in histatin-resistant derivatives of *Candida albicans*. FEMS Microbiol. Lett..

[44] Donlan R.M. (2002). Biofilms: Microbial life on surfaces. Emerg. Infect. Dis..

[45] Flemming H.C., van Hullebusch E.D., Neu T.R., Nielsen P.H., Seviour T., Stoodley P., Wingender J., Wuertz S. (2023). The biofilm matrix: Multitasking in a shared space. Nat. Rev. Microbiol..

[46] Lohse M.B., Gulati M., Johnson A.D., Nobile C.J. (2018). Development and regulation of single- and multi-species *Candida albicans* biofilms. Nat. Rev. Microbiol..

[47] Zare M., Ghomi E.R., Venkatraman P.D., Ramakrishna S. (2021). Silicone-based biomaterials for biomedical applications: Antimicrobial strategies and 3D printing technologies. J. Appl. Polym. Sci..

[48] Pierce C.G., Chaturvedi A.K., Lazzell A.L., Powell A.T., Saville S.P., McHardy S.F., Lopez-Ribot J.L. (2015). A novel small molecule inhibitor of *Candida albicans* biofilm formation, filamentation and virulence with low potential for the development of resistance. NPJ Biofilms Microbiomes.

[49] Lara H.H., Lopez-Ribot J.L. (2020). Inhibition of Mixed Biofilms of *Candida albicans* and Methicillin-Resistant *Staphylococcus aureus* by Positively Charged Silver Nanoparticles and Functionalized Silicone Elastomers. Pathogens.

[50] McConnell G., Rooney L.M., Sandison M.E., Hoskisson P.A., Baxter K.J. (2025). A simple silicone elastomer colonization model highlights complexities of *Candida albicans* and *Staphylococcus aureus* interactions in biofilm formation. J. Med. Microbiol..

[51] Kumpakha R., Gordon D.M. (2023). Occidiofungin inhibition of *Candida* biofilm formation on silicone elastomer surface. Microbiol. Spectr..

[52] Ceresa C., Tessarolo F., Maniglio D., Caola I., Nollo G., Rinaldi M., Letizia F. (2018). Inhibition of *Candida albicans* biofilm by lipopeptide AC7 coated medical-grade silicone in combination with farnesol. AIMS Bioeng..

[53] Kim K.S., Kim Y.S., Han I., Kim M.H., Jung M.H., Park H.K. (2011). Quantitative and qualitative analyses of the cell death process in *Candida albicans* treated by antifungal agents. PLoS ONE.

[54] Grela E., Zdybicka-Barabas A., Pawlikowska-Pawlega B., Cytrynska M., Wlodarczyk M., Grudzinski W., Luchowski R., Gruszecki W.I. (2019). Modes of the antibiotic activity of amphotericin B against *Candida albicans*. Sci. Rep..

[55] Gow N.A., Hube B. (2012). Importance of the *Candida albicans* cell wall during commensalism and infection. Curr. Opin. Microbiol..

[56] Garcia-Rubio R., de Oliveira H.C., Rivera J., Trevijano-Contador N. (2019). The Fungal Cell Wall: *Candida*, *Cryptococcus*, and *Aspergillus* Species. Front. Microbiol..

[57] Harrington B.J., Hageage G.J. (2003). Calcofluor White: A Review of its Uses and Applications in Clinical Mycology and Parasitology. Lab. Med..

[58] Sokol-Anderson M.L., Brajtburg J., Medoff G. (1986). Amphotericin B-induced oxidative damage and killing of *Candida albicans*. J. Infect. Dis..

[59] Mesa-Arango A.C., Trevijano-Contador N., Román E., Sánchez-Fresneda R., Casas C., Herrero E., Argüelles J.C., Pla J., Cuenca-Estrella M., Zaragoza O. (2014). The production of reactive oxygen species is a universal action mechanism of Amphotericin B against pathogenic yeasts and contributes to the fungicidal effect of this drug. Antimicrob. Agents Chemother..

[60] Mesa-Arango A.C., Scorzoni L., Zaragoza O. (2012). It only takes one to do many jobs: Amphotericin B as antifungal and immunomodulatory drug. Front. Microbiol..

[61] Anderson T.M., Clay M.C., Cioffi A.G., Diaz K.A., Hisao G.S., Tuttle M.D., Nieuwkoop A.J., Comellas G., Maryum N., Wang S. (2014). Amphotericin forms an extramembranous and fungicidal sterol sponge. Nat. Chem. Biol..

[62] Carolus H., Pierson S., Lagrou K., Van Dijck P. (2020). Amphotericin B and Other Polyenes-Discovery, Clinical Use, Mode of Action and Drug Resistance. J. Fungi.

[63] Dominguez E., Zarnowski R., Sanchez H., Covelli A.S., Westler W.M., Azadi P., Nett J., Mitchell A.P., Andes D.R. (2018). Conservation and Divergence in the *Candida* Species Biofilm Matrix Mannan-Glucan Complex Structure, Function, and Genetic Control. mBio.

[64] Mitchell K.F., Zarnowski R., Andes D.R. (2016). Fungal Super Glue: The Biofilm Matrix and Its Composition, Assembly, and Functions. PLoS Pathog..

[65] Nett J., Lincoln L., Marchillo K., Massey R., Holoyda K., Hoff B., VanHandel M., Andes D. (2007). Putative role of beta-1,3 glucans in *Candida albicans* biofilm resistance. Antimicrob. Agents Chemother..

[66] Tkacz J.S., Cybulska E.B., Lampen J.O. (1971). Specific staining of wall mannan in yeast cells with fluorescein-conjugated concanavalin A. J. Bacteriol..

[67] Ben-Ami R., Garcia-Effron G., Lewis R.E., Gamarra S., Leventakos K., Perlin D.S., Kontoyiannis D.P. (2011). Fitness and virulence costs of *Candida albicans FKS1* hot spot mutations associated with echinocandin resistance. J. Infect. Dis..

[68] Tan Y., Ma S., Leonhard M., Moser D., Schneider-Stickler B. (2018). β-1,3-glucanase disrupts biofilm formation and increases antifungal susceptibility of *Candida albicans* DAY185. Int. J. Biol. Macromol..

[69] Tan Y., Ma S., Ding T., Ludwig R., Lee J., Xu J. (2022). Enhancing the Antibiofilm Activity of β-1,3-Glucanase-Functionalized Nanoparticles Loaded with Amphotericin B Against *Candida albicans* Biofilm. Front. Microbiol..

[70] Xie Y., Hua H., Zhou P. (2022). Magnolol as a potent antifungal agent inhibits *Candida albicans* virulence factors via the PKC and Cek1 MAPK signaling pathways. Front. Cell. Infect. Microbiol..

[71] Qian W., Lu J., Gao C., Liu Q., Yao W., Wang T., Wang X., Wang Z. (2024). Isobavachalcone exhibits antifungal and antibiofilm effects against *C. albicans* by disrupting cell wall/membrane integrity and inducing apoptosis and autophagy. Front. Cell. Infect. Microbiol..

[72] Deveau A., Hogan D.A. (2011). Linking quorum sensing regulation and biofilm formation by *Candida albicans*. Methods Mol. Biol..

[73] Cleary I.A., Lazzell A.L., Monteagudo C., Thomas D.P., Saville S.P. (2012). *BRG1* and *NRG1* form a novel feedback circuit regulating *Candida albicans* hypha formation and virulence. Mol. Microbiol..

[74] Kim M.J., Cravener M., Solis N., Filler S.G., Mitchell A.P. (2024). A Brg1-Rme1 circuit in *Candida albicans* hyphal gene regulation. mBio.

[75] Qi W., Acosta-Zaldivar M., Flanagan P.R., Liu N.N., Jani N., Fierro J.F., Andrés M.T., Moran G.P., Köhler J.R. (2022). Stress- and metabolic responses of *Candida albicans* require Tor1 kinase N-terminal HEAT repeats. PLoS Pathog..

[76] Su C., Lu Y., Liu H. (2013). Reduced TOR signaling sustains hyphal development in *Candida albicans* by lowering Hog1 basal activity. Mol. Biol. Cell.

[77] Walraven C.J., Lee S.A. (2013). Antifungal lock therapy. Antimicrob. Agents Chemother..

[78] Kovács R., Majoros L. (2022). Antifungal lock therapy: An eternal promise or an effective alternative therapeutic approach?. Lett. Appl. Microbiol..

[79] Akkuş-Dağdeviren Z.B., Saleh A., Schöpf C., Truszkowska M., Bratschun-Khan D., Fürst A., Seybold A., Offterdinger M., Marx F., Bernkop-Schnürch A. (2023). Phosphatase-degradable nanoparticles: A game-changing approach for the delivery of antifungal proteins. J. Colloid. Interface Sci..

[80] Lombana A., Raja Z., Casale S., Pradier C.M., Foulon T., Ladram A., Humblot V. (2014). Temporin-SHa peptides grafted on gold surfaces display antibacterial activity. J. Pept. Sci..

[81] Oger P.C., Piesse C., Ladram A., Humblot V. (2019). Engineering of Antimicrobial Surfaces by Using Temporin Analogs to Tune the Biocidal/antiadhesive Effect. Molecules.

